# Supplementary material for: The Social Situation Affects How We Process Feedback About Our Actions
Source: Front Psychol. 2019 Feb 25;10:361. doi: 10.3389/fpsyg.2019.00361 (PMC6397836; doi:10.3389/fpsyg.2019.00361)
Supplement: Supplementary file 1 [file Data_Sheet_1.PDF]

frontiers

Supplementary Material: Article Title

1 INSTRUCTIONS

Here we present written instructions that were given to our participants. Additionally, the instructions were given orally with presentation of the task.

” This is a study on joint actions in different social conditions. Therefore you will perform the same task in two different contexts. In general, you will start the experiment with an initial budget of 10 euro, and depending on your performance you could increase (+5 cents) or decrease it (-5 cents) in each trial.

The task

You will be asked to perform a simple task. Specifically, you will be shown a target object (Gabor patch) consisting of two different features: degree of orientation and spatial frequency. From the beginning until the end of the experiment you will be responsible for only one of these two features, while your co-player will be in charge for the other one. First, you will see the target object in the middle of the screen, and after that four different objects will be displayed. Only one of them will have the same orientation or spatial frequency as the target. You will be asked to indicate, as soon as possible, which is the correct one (the same as the target). You will give your answer by button presses on the keyboard. You will have 3 seconds to give your answer. Afterwards feedback will appear: in green if your answer was correct, in red if you were incorrect.

Cooperation Condition

You will be performing the task in cooperation with your co-player. That means that your performance will be rewarded only in the case that both of you give the correct answers. In the opposite scenario, in which both of you will be incorrect that will cause a decrease of 5 cents from your budgets. In the other two cases (one of you correct while the other incorrect) the trial will end up in a tie and none will win or lose anything and your budget will be kept constant. The correctness of your answer and of your partner’s answer will be displayed in a circle feedback that it is divided in two parts (left and right). The part corresponding to your seat side will represent your response, while the other side refers to your co-player.

Competition Condition

In this condition you will compete with your co-player. Therefore, you will be rewarded only in the case that you are correct and the second player is incorrect. More clearly, you will gain the 5 cents only in case you gave the right answer and your co-player gave the wrong one. In the opposite scenario (you are incorrect, she/he is incorrect) you will lose 5 cents while the second player will gain 5 cents. In case of both correct or incorrect answers the trial will end up in a tie and none will lose or win anything. Feedback will be displayed in the same way as described above.

The experiment consists of a total of 16 blocks of 40 trials each. There will be breaks between blocks. Each block corresponds to a different social condition. It starts with information about the condition under which you are going to perform the task with short reminder of the rewarding for each possible feedback and it ends with a recap of the amount of your and your partner’s budget at the end of the given block. ”

2 SUPPLEMENTARY TABLES AND FIGURES

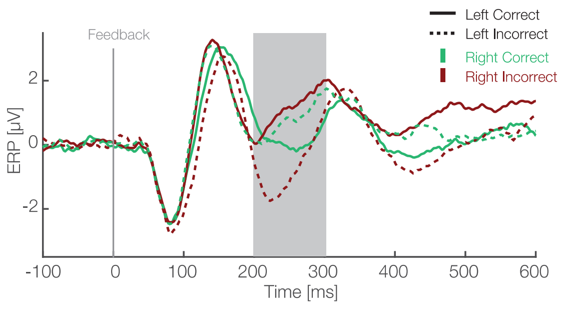

Figure S1. Cooperation. Feedback locked ERP waveforms at pooled electrode sites (F1, Fz, F2, FC1, FCz, FC2) in cooperation condition. Data are averaged referenced. Green and red colors represent the correct and incorrect trials of participants sitting on the right side. Solid and dashed lines represent correct and incorrect trials of participants sitting on the left side. The gray box shows the preselected time window used for the confirmatory statistical analysis (200-300 ms).

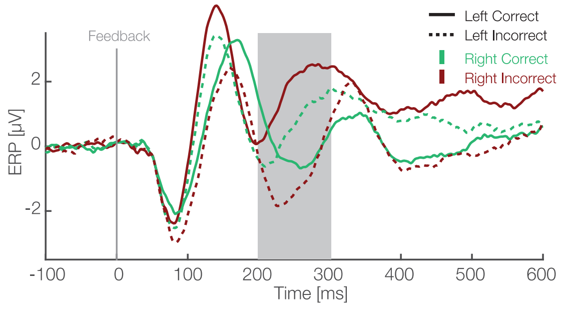

Figure S2. Competition. Feedback locked ERP waveforms at pooled electrode sites (F1, Fz, F2, FC1, FCz, FC2) in competition condition. Data are averaged referenced. Green and red colors represent the correct and incorrect trials of participants sitting on the right side. Solid and dashed lines represent correct and incorrect trials of participants sitting on the left side. The gray box shows the preselected time window used for the confirmatory statistical analysis (200-300 ms).
